# Clearing the Pathways to Self-Transcendence

**DOI:** 10.3389/fpsyg.2021.648381

**Published:** 2021-04-30

**Authors:** Piers Worth, Matthew D. Smith

**Affiliations:** Centre for Positive Psychology, Buckinghamshire New University, High Wycombe, United Kingdom

**Keywords:** self-transcendence, self-actualisation, COVID-19, flow, meaning

## Abstract

“Self-transcendence” is proposed as a way in which individuals might find relief and support in the context of COVID-19, as well as other times of uncertainty. However, the authors propose that the multiple definitions of self-transcendence within existing literature lean towards the complex, sometimes obscure, and imprecisely spiritual. A concern is that this creates a circumstance, where the possibility of supporting self-transcendence in a wider population will become excluding in this complexity. In this paper, we have undertaken a critical summary review focused primarily on historical foundations of the concept of self-transcendence, and key theoretical approaches in which self-transcendence and self-transcendent experiences are discussed with the motive of finding a clarity to understanding self-transcendence and the pathways towards it. We argue that this much-needed clarity in our understanding of self-transcendence may serve as an inclusive and democratized resource in which to support well-being and resilience in the context of COVID and beyond.

## Introduction

Wong et al. (2020, p. 1) make the case for a “new science” of self-transcendence (ST) as being central to our understanding of how humans might cope, even flourish, in the context of suffering and adversity such as that we are seeing in a world dealing with the COVID-19 global pandemic. They note that “self-transcendence involves a fundamental shift in one’s life attitude, from an egotistic focus to caring for others or something greater than oneself.”

In many ways, the “science” of ST has been a focus for psychologists since its inception with, for example, William James noting that “we can experience union with something larger than ourselves and in that union find our greatest peace” (James, 1902). More recently, self-transcendent experiences have been characterized as experiences that can span a spectrum of contexts and intensity where “the subjective sense of one’s self as an isolated entity can temporarily fade into an experience of unity with other people or one’s surroundings, involving the dissolution of boundaries between the sense of self and ‘other’ (Yaden, et al., 2017, p. 1).”

We, too, recognize the value of raising awareness and understanding of ST among researchers and the wider public alike, and it is with this aim in mind that we set out to clarify extant approaches to ST and highlight pathways towards it. We have done this in a sincere attempt to synthesize and, to some extent, simplify the writings of others to make the topic more accessible to a wider, non-technical, audience, and (a) support the reader in a broader conceptual understanding of ST and (b) make the possibility of experiencing ST more accessible. The “pathways” to ST we explore include: ST through “being human”; ST through “meaning”; ST through “self-actualisation”; ST through “flow”; ST through the “life span.” We further summarize attempts to theoretically model ST. When brought together, we argue, the pathways towards ST become clearer and more navigable. It is our hope that is this short review stimulates others to explore the wider literature what is a clearer sense of direction, and of how psychologists, in particular, have addressed the topic of self-transcendence.

## Self-Transcendence Through “Being Human”

Two of the earlier writers to discuss self-transcendence are Frankl and May, pioneers of existential psychology (e.g., Frankl, 1946/2020, 1959/1992, 1966; May et al., 1960; May, 1983). Both Frankl and May drew attention to the very act of being human involved a form of self-transcendence in that to exist, or to be, involves our attention being directed outside of ourselves towards other people and the world at large. This perspective on self-transcendence appears to be somewhat different from what William James alludes to, and indeed the majority of “self-transcendent experiences” explored in detail by Yaden and colleagues (James, 1902; Yaden et al., 2017).

May et al. (1960) and May (1983) defines “being” as the propensities within us that are continually emerging and becoming, that will stand out in time. He proposes that our self-awareness as human beings expands the range of our consciousness and thereby creates the situation in which we may transcend our immediate circumstances. He argues, further, our self-consciousness or self-awareness makes the capacity for self-transcendence as inseparable through the way in which we might reflectively stand outside a situation and consider its content and characteristics as well as choices for the future. In exploring both the characteristics of psychological existence and highlighting the vibrancy with which we come into “being” as a constant process, May has described self-transcendence in a manner that is built upon by subsequent writers.

## Self-Transcendence Through “Meaning”

Frankl’s approach, in particular, emphasizes the centrality of what he refers to as humans’ “will to meaning,” in that the very nature of being human involves the need to seek and create meaning as a fundamental force or aspect of human nature. It is what it means to be human. In order to survive, even flourish, that is to be fully human, we must make sense of our existence in the world (Frankl, 1946/2020, 1966). Frankl argues that our existence is not authentic unless it involves the self-transcendent quality of this form of attention, beyond us and towards meaning. Life questions us, and in the act of finding meaning we are taking responsibility for our own lives.

It is proposed that we can find meaning in our life in three possible ways (Frankl, 1959/1992). First, in the creation of work or a deed or action. Second, through the experience of something or someone. Third, by the attitude we choose to adopt when we face unavoidable suffering (which he defines as “fate”). His writing proposes suffering is only one of three possible sources of meaning, yet unsurprisingly given the intensity of his own wartime experiences, he offers profound examples of how this may occur through a transformation of the attitude we hold towards the circumstance (Frankl, 1946/2020). Frankl was specific that we have a spiritual dimension as part of our being, that it may be theistic or non-theistic, and in our finding personal meaning and/or supporting others doing so, this dimension warranted recognition, and that its presence and development was a reflection of our wholeness as individuals (Frankl, 1969/2004).

## Self-Transcendence Through “Self-Actualisation”

Maslow’s awareness of self-transcendence grew from his work on self-actualisation, and the reports that came from self-actualising individuals of “peak experiences” (e.g., Maslow, 1964, 1968). Maslow (1968) defined self-actualisation in four parts: the actualisation of our talents, capacities, and potential; which in turn enabled a fulfillment of what we may perceive as our “mission” or vocation; which in turn involves a deeper understanding of our own nature; and creates a trend in which we move towards personal integration and unity.

“Peak” experiences were one of the characteristics of self-actualising individuals identified by Maslow which he defined as wonderful experiences, rapturous, and ecstatic moments (Maslow, 1968, 1970). In these experiences, individuals would appear to become self-forgetful, unselfish, and ego transcending (Maslow, 1964). An integration or unity within their being was experienced, and between the individual and their sense of the world around them. Maslow also wrote of a transcendence of the self in creative work, in which an oneness with the work undertaken occurred that could be felt as ecstasy and exaltation (Maslow, 1971). This form of self-transcendence may involve a profound sense of absorption, sense of loss in time, giving up the past and the future, and a narrowing of consciousness into present time and work.

Maslow expressed surprise in this work at the presence of transcendence in self-actualisers, perceiving the effort that was invested into developing their own potential, he acknowledged, that they then, in turn, invested time in the support of others. He proposed that self-actualisers were commonly invested in a cause outside or beyond themselves. A comment that recurs in Maslow’s writing likens those displaying ST as showing the “Bodhisattva” state, oriented to support and to serve those beyond themselves in difficulty or confusion (Hoffman, 1999). This reflects an apparent paradox in that individuals who invest in or are motivated by the development of their potential, in becoming a healthy and strong “ego,” in turn then seek to let go of this state to merge with causes and the need for support of other individuals or causes (Maslow, 1971). Kaufman (2020) proposes that self-actualisation acts as a “bridge” to self-transcendent states, values, and motivation. Maslow (1964) proposed that, in transcending polarities and dichotomies in life experience, we open to a broader and more accepting perception of ourselves and the world around us.

## Self-Transcendence Through “Flow”

The experience of a form of “transcendence” of the self in creative work identified by Maslow has been studied in depth by Csikszentmihalyi (1992). Csikszentmihalyi, guided by those he interviewed, termed this experience “flow,” and noted it would typically occur when we accept an opportunity and challenge to act that is on the edge of our current expertise. The challenges we are oriented towards will be a reflection of our talents, strengths, and values. The task needs focus and attention that is likely to absorb our capacity for attention, which, in turn, results in a decrease in awareness of self and absorption in time – the boundaries of the self-relax and become absorbed in an outer experience. Csikszentmihalyi sees polarities occurring in these experiences: moving from the known to the unknown, focusing attention on the outer experience, which decreases inner an awareness of self and time. He proposes that if there is a willingness to enter this state of growth and development, over time, we incrementally develop our uniqueness and, in the transcendence, become oriented towards the outer goals that contribute to others.

## Self-Transcendence Through “The Lifespan”

Life-span development theories point to a process of self-transcendence as we age. For example, the Erikson and Erikson’s theory of bio-psycho-social life span development (e.g., Erikson, 1951; Erikson and Erikson, 1997) suggest life comprises eight (subsequently revised to nine) stages of growth characterized by a polarity tension between two potential outcomes ideally leading the emergence, on balance, of a developmental strength. Further that the outcome of each stage would be revisited and revised cumulatively in each subsequent stage. This conveys how an individual may adjust, change, deepen their sense of self, and the life perception repeatedly through life, an interpretation put forward by Erikson and Erikson and reflecting McAdams (1993) research that we construct and adjust our sense of self and life story through time.

Reed (2018) proposes that ST is a life perspective that arises in developmentally maturing experiences, through aging or other life experiences at any age that advance awareness of mortality and value of life. She suggests that this form of cognition will not seek absolute answers and in turn will seek meaning in life experiences, integrating this in a broader social, moral, and historical context. Core to Reed’s theory is that we are integrally connected to our environments and that the experience of self-transcendence connects us to our self, others, and our environment. Reed defines self-transcendence as an expansion of our boundaries of our self: (i) intra-personally (towards a greater awareness of our inner state, values, and dreams); (ii) interpersonally (supporting the way in which we relate to others and our environment); (iii) temporally (through which we integrate our sense of past and future that alters meaning in our present; and (iv) trans-personally (through which we connect dimensions beyond the discernible world). In related work, McCarthy and Bockweg (2013) undertake an extensive concept analysis and make a strong case for five domains of transcendence: relationships, creativity, contemplation, introspection, and spirituality.

Similarly, Tornstam has been reformulating and deepening our understanding of the experience of aging, noting that, beyond the losses of aging, there is still a capacity to learn, change, and psychologically grow (e.g., Tornstam, 1997, 2011). His concept of “gerotranscendence” encapsulates the transcending of “borders and barriers” of earlier life experiences that is multidimensional (cosmic, the self, and social and personal relationships).

In both Tornstam’s research and the Eriksons’ theories, the process of aging and transcendence is viewed as a developmental process towards a higher state of personal maturity. Indeed, Tornstam is overt about being inspired by, among others, the work of Jung (1930) who proposed within us the process of “individuation,” potentially becoming the whole and complete individual we are. Further, as part of that unfolding, Jung theorized what he named as “the transcendent function,” a process in which the polarities of life experience, conscious, and unconscious, were reconciled and in turn influenced the development of human consciousness in ways illustrated by Tornstam’s research (Stein, 2006/2018).

## Three Steps To Self-Transcendence

Wong has further modeled self-transcendence, building directly on the work of Frankl (e.g., Wong, 2016). Wong proposes meaning-seeking and self-transcendence are a fundamental expression of our spiritual nature and, through this, influence our healing and well-being. He suggests that the more we “forget ourselves” in the act of giving ourselves to a cause, service, or love, the more human we become, and, in turn, actualise who we truly are. Wong dismisses “self-actualisation” in its own right and believes that it is simply a “side effect” of self-transcendence.

For those of us seeking self-transcendence, Wong helpfully proposes three levels that offer a sequenced, unfolding focus on what this might involve:

1. Seeking situational meaning: this involves looking beyond our personal or situational constraints to values, which may be spiritual. To do this, we would rely on mindfulness of the present moment in our inner and outer experiences and the need to maintain an attitude of “openness, curiosity, and compassion.”
2. Seeking our calling: through this, we seek and pursue and engage with a higher purpose, mission, or vocation connected to or serving a greater good. This may have the characteristics of concrete meaning or life goals in the direct service of others. Wong proposes that calling is not only about work or career, but also how we respond to the demands of life itself. Our expression or response to our calling would draw upon the uniqueness of our talents, our personal temperament or experiences.
3. Seeking ultimate meaning: to look beyond the current context, the physical limitations we experience, and time and space, to a transcendental realm. It is here, Wong helpfully recognizes that not everyone focuses on a religiously-oriented spirituality and defines what non-theistic seekers might consider, such as “ideas of goodness, truth and beauty.” This level of transcendence will reflect an individual’s assumptions about the world, philosophy, views, and beliefs.

**Figure 1:**
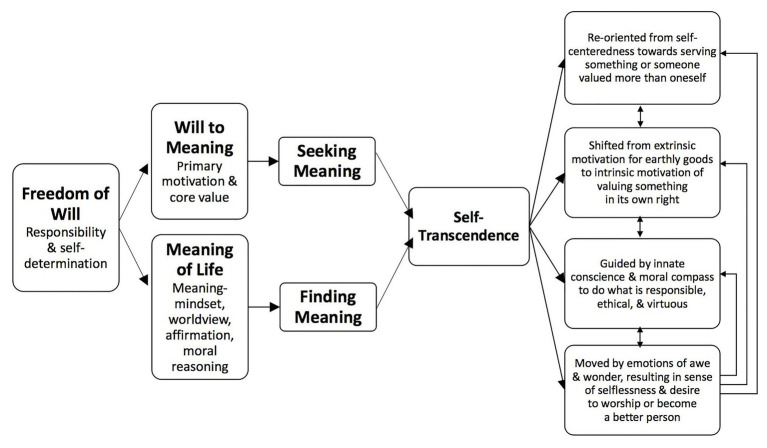
Frankl’s Model of Self-Transcendence (Wong and Reilly, 2017) - used with permission.

Wong (2016) suggests that self-transcendence at each of these levels will involve a continuous process of personal improvement in order to expand our potential. He emphasizes that this improvement process is not based on self-reference, but based on service to others. Wong and Reilly (2017) builds on the earlier paper and develops an articulate and helpful diagrammatic pathway to self-transcendence emerging from Frankl’s and his own theorizing (see **Figure 1** below).

The bedrock of this model is Frankl’s “freedom of will” as a central aspect of human existence in which we would respond ethically and responsibly towards others and the demands of life on us. Wong quotes Frankl’s term “response-abilit” as a focus of our capacity as human beings to respond thoughtfully and morally to the experiences we encounter, and through this we have the ability for self-determination. Two qualities and characteristics emerge from freedom of will. First, it is the “will to meaning” proposed by Frankl. This is the orientation, motivation, and energy to seek meaning. If we have a will to meaning, it follows within Wong’s theory that we would be seeking, finding, and experiencing “meaning of life.” In Wong’s theory, this is operationalized by a “meaning mindset” that enables us to find meaning and respond constructively to our experiences.

Wong proposes that the “will to meaning” is a motivational factor, and the meaning mindset is a cognitive capacity or response to identify and discover meaning in life. He believes these two factors lead to a discovery and experience of self-transcendence. Wong identifies characteristics of self-transcendence as a shift in focus from self to other, a shift in our values from extrinsic to intrinsic, an increase in moral concern, and the experience of elevated emotions such as awe or ecstasy. Going further, Wong suggests that these experiences produce a spiral effect in which meaning, virtue, and happiness interact and cumulatively build.

## Conclusion

This summary review was intentionally a sense-making process. By drawing out the themes that are apparent across the approaches reviewed, we can explore the most effective ways these can be integrated. For example, historically there have differences of opinion regarding the relationship between “self-actualisation” (as articulated by Maslow) and “self-transcendence” (as articulated, for example, by Frankl and more recently Wong). Some of these differences can be reconciled to some degree by adopting the “both/and” perspective instead of an “either/or” perspective. Indeed, being fully human, is a “both/and” experience and in taking this perspective some of the common ground between theories becomes apparent. We transcend ourselves in becoming ourselves, and we transcend ourselves through giving our self, and in the giving also actualise our potential. Self-transcendence occurs in and through self-actualisation in the development of our potentialities, in which the experienced boundaries between self and other dissolve. In this way, we echo Csikszentmihalyi (1993) when he proposes that we proceed in the phases of differentiation and integration and the dialectical spiral pathways of development.

This “both/and” perspective is also apparent in recognizing self-transcendence can be experienced and observed as a localized outcome of our approach to a task (e.g., as in “flow”) or small acts that become cumulative and developmental *over time and* as a process of cognition at a time of life in illness and approaching death (as in the contexts pertinent to the work of Reed, Erikson, Tornstam, and McCarthy and Bokweg). Self-transcendent experiences exist on a spectrum of possibility, intensity, and choice (Yaden et al., 2017). If viewed in this way, we see more shared ground than difference.

### Prompts Towards Self-Transcendence

Our motive was to offer a compass-bearing on psychological approaches to self-transcendence to a wider audience in the face of demands and impact of experiences of COVID. With this in mind, we end with some prompts to stimulate the personal exploration of self-transcendence in the face of uncertainty that come from our own learning from the literature. They are offered with openness, curiosity, and compassion:

- Seek initially to stand back from the context and ask yourself how specifically you might contribute and be challenged in what you face?
- What positive life-oriented meaning might be found in this context? Might this be an internal perspective for ourself or externally in terms of personal experience and contribution?
- What personal development or actualising of your potential does this situation ask of you? Are you in a circumstance where you are able and wish to be in service to others? How can you bring this about?
- What polarity of positive intention and situational challenge are you likely to face in this circumstance? In what way can you bring about your positive intent while also caring for the pressure and strain you may face?
- Are you willing to identify and engage with tasks or activities that balance your challenges and skills, and in the absorption in the work allow yourself to be in flow, learning, and developing while you also give to others?
- How might you maintain your attention mindfully in your experiences, considering the needs of large-scale difficulties as well as the contribution that may be made in small acts cumulatively in working in this context?
- How willing are you to allow events to unfold in a way, where you relinquish control to something greater? How might you “trust the process”?

## Author Contributions

All authors listed have made a substantial, direct and intellectual contribution to the work, and approved it for publication.

### Conflict of Interest

The authors declare that the research was conducted in the absence of any commercial or financial relationships that could be construed as a potential conflict of interest.
